# Comparison of Efficacy and Toxicity of Traditional Chinese Medicine (TCM) Herbal Mixture LQ and Conventional Chemotherapy on Lung Cancer Metastasis and Survival in Mouse Models

**DOI:** 10.1371/journal.pone.0109814

**Published:** 2014-10-06

**Authors:** Lei Zhang, Chengyu Wu, Yong Zhang, Fang Liu, Xiaoen Wang, Ming Zhao, Robert M. Hoffman

**Affiliations:** 1 AntiCancer, Inc., San Diego, California, United States of America; 2 Department of Traditional Chinese Medicine Diagnostics, Nanjing University of Chinese Medicine, Nanjing, China; 3 Department of Surgery, University of California San Diego, San Diego, California, United States of America; 4 Department of Anatomy, Second Military Medical University, Shanghai, China; The First Affiliated Hospital of Nanjing Medical University, China

## Abstract

Unlike Western medicine that generally uses purified compounds and aims to target a single molecule or pathway, traditional Chinese medicine (TCM) compositions usually comprise multiple herbs and components that are necessary for efficacy. Despite the very long-time and wide-spread use of TCM, there are very few direct comparisons of TCM and standard cytotoxic chemotherapy. In the present report, we compared the efficacy of the TCM herbal mixture LQ against lung cancer in mouse models with doxorubicin (DOX) and cyclophosphamide (CTX). LQ inhibited tumor size and weight measured directly as well as by fluorescent-protein imaging in subcutaneous, orthotopic, spontaneous experimental metastasis and angiogenesis mouse models of lung cancer. LQ was efficacious against primary and metastatic lung cancer without weight loss and organ toxicity. In contrast, CTX and DOX, although efficacious in the lung cancer models caused significant weight loss, and organ toxicity. LQ also had anti-angiogenic activity as observed in lung tumors growing in nestin-driven green fluorescent protein (ND-GFP) transgenic nude mice, which selectively express GFP in nascent blood vessels. Survival of tumor-bearing mice was also prolonged by LQ, comparable to DOX. *In vitro*, lung cancer cells were killed by LQ as observed by time-lapse imaging, comparable to cisplatinum. LQ was more potent to induce cell death on cancer cell lines than normal cell lines unlike cytotoxic chemotherapy. The results indicate that LQ has non-toxic efficacy against metastatic lung cancer.

## Introduction

Lung cancer is the leading cause of cancer death with non–small-cell lung cancer (NSCLC) accounting for approximately 80% of thoracic malignancies. The overall 5 years survival for NSCLC remains poor, approximately 16%. Lung cancer has shown little improvement in survival for the last 30 years [1]. Novel approaches to the treatment of lung cancer are urgently required. Nature products are important resources for drug development. For cancer disease, 60% of new drugs, originate from natural sources. There is currently increasing interest in traditional Chinese medicine (TCM) herbal mixtures which have been used to treat cancer for thousands of years in China. Unlike Western medicine that generally uses purified compounds and aims to target a single molecule or pathway, TCM compositions usually comprise multiple herbs and components. There is much anecdotal evidence of the efficacy of TCM in the form of herbal mixtures in cancer patients [2]–[9].

In a previous study, we determined the inhibitory efficacy of the TCM herb Celastrus orbiculatus Thunb. (COT) on tumor growth, metastasis and antiogenesis of hepatocelluar carcinoma (HCC) Hep-G2 cells in an orthotopic nude mouse model using fluorescence imaging technology. Whole-body fluorescence imaging was performed to measure tumor growth and monitor metastasis development. High-dose, early treatment with COT demonstrated significant efficacy on controlling tumor volume and tumor weight in the human HCC Hep-G2 orthotopic tumor model [4].

The efficacy of the TCM herb tubeimu, extracted from the tuber of the plant *Bolbostemma paniculatum* was tested on MDA-MB-231 human breast cancer cells *in vitro*. The MDA-MB-231 cell line was engineered to express RFP in the cytoplasm and GFP linked to histone H2B in the nucleus, which allows real-time imaging of nuclear-cytoplasmic dynamics. Apoptosis was readily visualized in these cells by nuclear shape changes and fragmentation. The MDA-MB-231 RFP-GFP cells were cultured either in two-dimensions on plastic or in three dimensions on Gelfoam. Cells were treated with a dichloromethane extract of fresh tubeimu. Tubeimu induced apoptosis of MDA-MB-231 cells, as observed by fluorescence microscopy, as early as 24 hours of treatment in vitro in two-dimensional culture. By 48 hours’ treatment, DNA fragmentation could be observed. Tubeimu also induced apoptosis of MDA-MB-231 cells in three-dimensional culture on Gelfoam, but to a lesser extent than in 2D culture [5].

Despite the very long-history and wide-spread use of TCM, there are very few direct comparisons of TCM and standard cytotoxic chemotherapy. We have previously developed a TCM formulation of herbs, LQ, that has been shown to have potent non-toxic therapeutic properties in clinically and in orthotopic mouse models of pancreatic cancer [6]. We compared LQ to gemcitabine, which is first-line therapy for pancreatic cancer for anti-metastatic and anti-tumor activity as well as safety. The therapeutic efficacy of LQ was comparable with gemcitabine but with less toxicity [6].

In the present report, we used state-of-art fluorescence imaging technology and animal models to compare efficacy and toxicity of LQ to doxorubicin (DOX) and cyclophosphamide (CTX) on lung cancer. With the use of GFP and/or RFP stably expressed in human cancer cells or mice, the efficacy of LQ was compared to standard chemotherapy on tumor and metastatic growth as well as angiogenesis.

We demonstrate here that LQ has antitumor, anti-metastatic and anti-angiogenic efficacy in clinically-relevant mouse models of lung cancer comparable to doxorubicin and cyclophosphamide, without their toxicity.

## Materials and Methods

### Ethics statement

All animal studies were conducted with an AntiCancer Institutional Animal Care and Use Committee (IACUC)-protocol specifically approved for this study and in accordance with the principals and procedures outlined in the National Institute of Health Guide for the Care and Use of Animals under Assurance Number A3873-1. In order to minimize any suffering of the animals the use of anesthesia and analgesics were used for all surgical experiments. Animals were anesthetized with a 20 µL mixture of Ketamine (22–44 mg/kg), Acepromazine (0.75 mg/kg), and Xylazine (2–5 mg/kg) by intramuscular injection 10 minutes before surgery. The response of animals during surgery was monitored to ensure adequate depth of anesthesia. Ibuprofen (7.5 mg/kg orally in drinking water every 24 hours for 7 days post-surgery) was used in order to provide analgesia post-operatively in the surgically-treated animals. The animals were observed on a daily basis and humanely sacrificed by CO_2_ inhalation when they met the following humane endpoint criteria: prostration, skin lesions, significant body weight loss, difficulty breathing, epistaxis, rotational motion and body temperature drop. The use of animals was necessary to understand the in vivo efficacy, in particular, anti-metastatic efficacy of the agents tested. Animals were housed with no more than 5 per cage. Animals were housed in a barrier facility on a high efficiency particulate air (HEPA)-filtered rack under standard conditions of 12-hour light/dark cycles. The animals were fed an autoclaved laboratory rodent diet.

### Cell lines and cell culture

Human lung cancer cell lines H460 [12], A549 [13], mouse Lewis lung carcinoma (LLC) [14], and monkey normal kidney endothelial cell line (VERO) [15] were maintained in RPMI-1640 (HyClone, South Logan, UT) with 10% fetal bovine serum (Gemini Bio-Products, Calabasas, CA). Human umbilical vein endothelial cells (HUVEC) [16] was maintained in EGM-2 Bulletkit medium (Lonza, Anaheim, CA).

LLC cells stably expressed red fluorescent protein (RFP), as previously described [10], [11]. H460 cells stably expressed green fluorescent protein (GFP), as previously described [12], [17]–[19]. H460 dual-color cells expressed GFP linked to histone H2B in the nucleus as previously described [20]–[22].

### Mice

Athymic nude mice (*nu/nu*) and C57BL/6 mice (AntiCancer Inc., San Diego, CA), 6–8 weeks old, were used in this study. Nestin-driven-GFP (ND-GFP) transgenic C57/B6 nude mice (AntiCancer, Inc.) expressing GFP under control of the nestin promoter were also used [23]–[26]. Non-transgenic C57 B/6 mice (AntiCancer, Inc.) were also used.

### Subcutaneous tumor growth

A549, H460, LLC, and H460-GFP cells were harvested by trypzinization and washed two times with phosphate-buffered saline (PBS) (HyClone, South Logan, UT). Cells (5×10^6^) were injected subcutaneously into the right flank of mice in a total volume of 100 µl PBS within 30 min of harvesting. The subcutaneous tumors were also used as the source of tissue for orthotopic implantation into the lung.

### Surgical orthotopic implantation (SOI)

Tumor pieces (1 mm^3^) derived from H460-GFP subcutaneous tumors growing in the nude mouse were implanted by surgical orthotopic implantation (SOI) [27] onto the left visceral pleura in cohorts of additional nude mice [12], [28]–[30]. The mice were anesthetized by Isofluran inhalation. A small 1 cm transverse incision was made on the left-lateral chest of the nude mice via the fourth intercostal space. A small incision (0.4–0.5 cm) between the third and fourth rib on the chest wall provided access to the pleural space and resulted in total lung collapse. Tumor fragments (1 mm^3^) were sewn together with an 8-0 surgical suture and fixed by making one knot. The lung was taken up by forceps and the tumor fragment sewn into the lower part of the lung with one suture. The lung tissue was then returned into the chest cavity. The chest muscles and skin were closed with a 6-0 surgical suture. The closed condition of the chest wall was examined immediately and, if a leak existed, it was closed by additional sutures. After closing the chest wall, the lung was un-inflated by withdrawing air from the chest cavity with a 25-gauge ½ needle. After the withdrawal of air, a completely inflated lung can be seen through the thin chest wall of the mouse. Then, the skin and chest muscle were closed with a 6-0 surgical suture in one layer. All procedures of the operation described above were performed with a 7× microscope [12].

### Experimental metastasis syngeneic mouse model

LLC-RFP cells (2×10^6^) were harvested by trypsinization and washed with cold PBS, then injected in the tail vein of nude or C57B/6 mice in a total volume of 100 µl with a 1 ml 29-gauge, latex-free syringe within 30 minutes of harvesting. The seeding and arrest of single cancer cells on the lung, accumulation of cancer-cell emboli, cancer-cell viability, and metastatic colony formation were imaged at 14 days after cell injection [31]. Individual images of excised lungs from the mice in the experimental metastasis model were obtained with the OV100 Small Animal Imaging System (Olympus Corp., Tokyo, Japan). The total red fluorescence area and lung weight were measured for metastatic burden. The images were analyzed using Imaging-Pro plus 6.0 software (MediaCybernetics Inc., Rockville, MD).

### Color-coded angiogenesis model

ND-GFP transgenic nude mice, 6 to 8 weeks old, were used. The mice were anesthetized and RFP-expressing LLC cells (5×10^5^ cells in 25 µl) were injected into the skin of the ear and footpad of the ND-GFP nude mice with a 1 ml 27G½ latex-free syringe [32]. The total length of GFP-expressing blood vessels and fluorescence intensity were quantitated. The images were analyzed with Imaging-Pro plus 6.0 software (MediaCybernetics Inc.).

### Assessment of efficacy *in vivo*

Efficacy of treatment was determined by standard measurements of tumor volume and tumor weight in the subcutaneous models. Tumor volume was calculated using the formula (long diameter×short diameter^2^)/2. In the orthotopic models, mice were sacrificed and explored when they appeared pre-morbid. After euthanasia, each mouse underwent laparotomy and median sternotomy and was then imaged in order to identify primary and metastatic tumors by imaging RFP or GFP fluorescence. The OV100 Small Animal Imaging System was used [33]. After performing full-body, open imaging, the solid organs were thoroughly examined for any evidence of metastasis. Body weight, organ structure observation, and general appearance of each mouse were monitored and recorded as evidence of systemic toxicity.

### Haematoxylin and Eosin (H&E) staining

The heart, liver, spleen, lung, kidney, and intestine of mice from each group were collected and embedded in frozen tissue matrix (OCT) and frozen immediately with liquid nitrogen. All the tissues were prepared as frozen sections and cut into 5 µm-thick slides for H&E staining. The histopathological structures of different organs were examined using an Olympus BH-2 microscope.

### Preparation of crude extracts of Chinese herbs

LQ is a mixture of Chinese medicinal herbs, comprising *Sinapis alba, Atractylodes macrocephala, Coix lacryma-jobi*, and *Polyporus adusta* and prepared at the School of Pharmacy, Nanjing University of Traditional Chinese Medicine as previously reported [6]. The ratio used (2∶3∶4∶3) is from dried plants. The plant parts used were *Sinapis albe:* seed; *Atractylodes macrocephala:* root; *Coix lacryma-jobi:* kernel; *Polyporus adusta*: whole mushroom. We followed US Pharmacopoeias UPS231 for heavy metal testing. All the herbs were tested and the levels of heavy metal were below the US Pharmacopoeias minimum daily dose. Each herb was extracted in boiling water for 20 mins, the solution was filtered. The residue was extracted with 75% ethanol, and the extract was filtered. Both the water extract and ethanolic extract were combined and concentrated by lyophilization. To obtain 100 mg of LQ required 402 mg of dried herbs. The lyophilized powder was suspended in PBS. The mixture was vortexed for 1 min and incubated at 80°C for 30 min. The sample was cooled to room temperature and was then centrifuged at 2000 rpm for 10 min. The supernatant was collected at a final concentration to 90 mg/ml. LQ was then diluted for dose-ranging experiments in the mouse models or filtered through a 0.2 µm membrane for *in vitro* use. *In vivo* dosing was by gavage.

### Drugs

Doxorobucin (DOX) (Bedford Laboratories, Bedford, OH), cyclophosphonate (CTX) (SIGMA, St. Louis, MO), Pingxiao (PX) (C.P. Pharmaceutical Co., Ltd., Xian, China), cisplatinum (CDDP) (DONG-A Pharmaceutical Co., Seoul, Korea), and paclitaxel (Taxol) (Bedford Laboratories, Bedford, OH) were used.

### Time-lapse imaging of H460 dual-color cells

A FluoView FV1000 confocal laser microscope (Olympus Corp., Tokyo, Japan) was used to image H460 dual-color cells treated with LQ and CDDP for time-lapse imaging. High-resolution images were captured directly for a 72-hour period with 30 min intervals at 37°C.

### MTS assay

The cells were exposed to various concentrations of LQ and chemotherapeutic agents for 72 hours. The number of viable cells was subsequently determined using the Cell Titer 96 Aqueous One Solution Cell Proliferation assay (Promega, Madison, WI) as described in the instructions.

### Statistical analysis

Data were assessed using the Student’s *t*-test. Kaplan-Meier analysis with a log-rank test was used to determine survival and differences between control and treatment groups. A *p* value of ≤0.05 was defined as statistically significant.

## Results and Discussion

### Comparison of efficacy of LQ and CTX on subcutaneous mouse models of lung cancer

**Figure 1:**
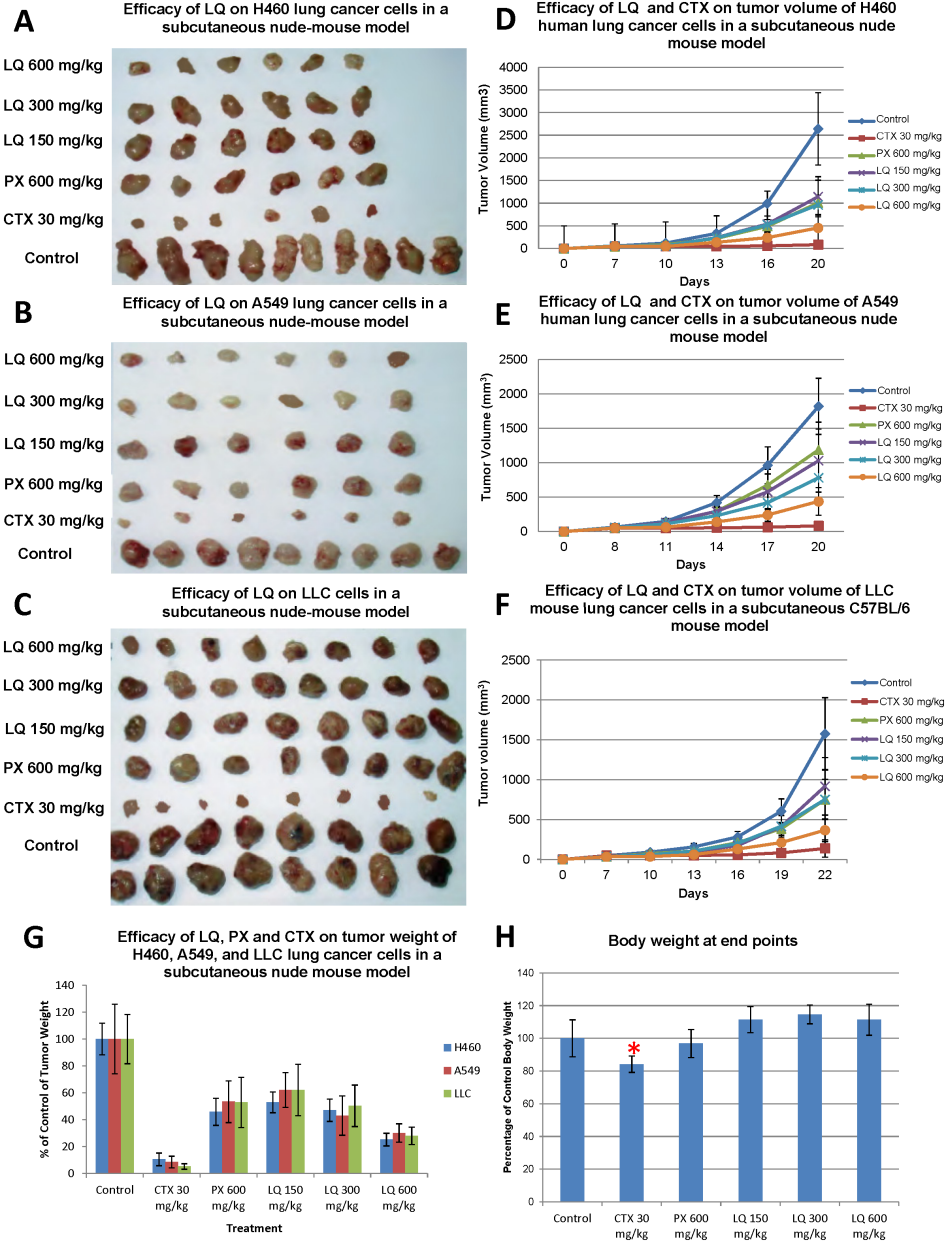
Efficacy of LQ on tumor size, growth and weight in subcutaneous nude mouse tumor models. For the H460 and A549 cell lines, each treatment group contained 6 mice and 10 mice were used for the untreated control groups. For the LLC cell line, each treatment group contained 8 mice and 16 mice were used for the untreated control group. After the subcutaneous tumors grew, the nude mice were given either CTX (30 mg/kg/day i.p.) for 7 days or PX (600 mg/kg/day p.o) for 10 days. PBS (po) was used in the control group. Mice were treated at 150, 300, and 600 mg/kg/day of LQ (po) for 10 days. Tumor size was measured twice a week. Tumor weight was measured at the endpoint. Statistical significance between groups was determined with the Student’s *t*-test. H460 (**A&D**), A549 (**B&E**), and LLC (**C&F**) had growth inhibition and tumor size inhibition after treatment with all agents. The tumor weight was also significantly inhibited by all agents (**G**) (*p<*0.01). After LQ treatment, lung tumor growth and tumor weight were significantly inhibited, compared to the untreated control group in all models (*p<*0.01). CTX and PX treatment resulted in a significant inhibition of tumor weight (*P<*0.01) compared to the control group for all cell lines. LQ had more efficacy than PX (*p<*0.05) on all cell lines (**G**). CTX induced loss of body weight (*p<*0.05), but not LQ or PX (**H**).

We tested the efficacy of different dosages of LQ and CTX on different lung cancer cell lines *in vivo*. H460, A549, and LLC cells growing subcutaneously for 7 days in nude mice were treated with either LQ (150, 300, and 600 mg/kg) for 10 days, the TCM herbal mixture PX [34] (600 mg/kg, daily gavage) for 10 days, or CTX (30 mg/kg/dose daily i.p) for 7 days. In the control group, mice were given PBS by oral administration. Tumor growth measured by size was significantly inhibited by LQ at doses of 150 mg/kg, 300 mg/kg, and 600 mg/kg in a dose-dependent manner (Figure 1A–F) (all p<0.01). Final tumor weight was also significantly decreased after LQ treatment at all dosages (all p<0.01) (Figure 1G). CTX and PX also inhibited tumor size and tumor weight significantly, compared to the control mice (all p<0.01) (Fig. 1A–G). LQ (600 mg/kg) was more effective than PX (600 mg/kg) based on tumor size and weight (all p<0.01) (Fig. 1D, E, F and G).

**Figure 2:**
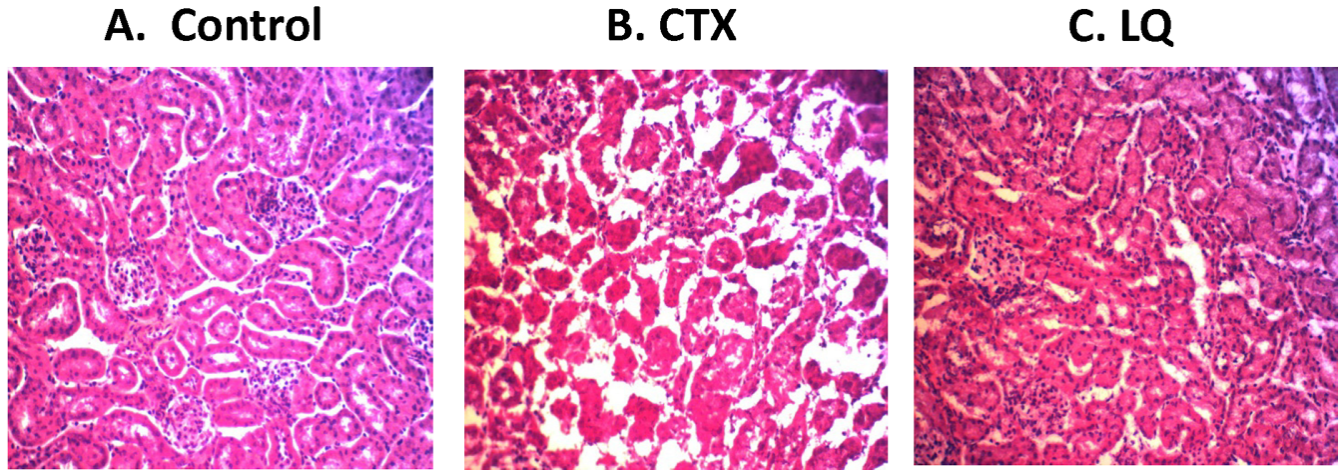
Toxicity of treatment on the renal cortex. (A) Renal cortex of untreated mouse. (B) The renal cortex of mice treated with CTX demonstrated renal toxicity. Toxicity was mainly in the proximal tubular cells, including degeneration, obstruction, necrosis and swelling. (C) The renal cortex of mice treated with LQ had no toxicity.

The body weight and general appearance of each mouse was observed during the experiments and at the end point. The body weight in CTX-treated mice significantly decreased after treatment (*p<*0.05) and recovered slowly after treatment termination. However, there was no decrease on body weight in LQ groups with all dosages. (Fig. 1H). The heart, liver, spleen, lung, kidney, and intestine of a mouse from each group (untreated control, LQ and CTX) were stained with H&E to visualize the toxicity after treatment. The histological structure of each organ was observed and compared microscopically. Figure 2 shows morphological changes in the renal tissue after CTX administration. After CTX treatment, the proximal tubule cells were injured, including swelling, degeneration, and necrosis and sloughing of tubular epithelial cells (Fig. 2B). However, the changes in renal tissue were not observed in LQ treated mice (Fig. 2C). Those results indicated that renal toxicity was caused by CTX, but not LQ.

The purpose of this experiment was to compare LQ to conventional chemotherapy at published therapeutic effective doses (ED) [35]. A low dose of doxorubicin was also tested which did not have apparent toxicity but had less efficacy compared to LQ (data not shown).

### Comparison of LQ and DOX on the H460-GFP human lung cancer subcutaneous mouse model

**Figure 3:**
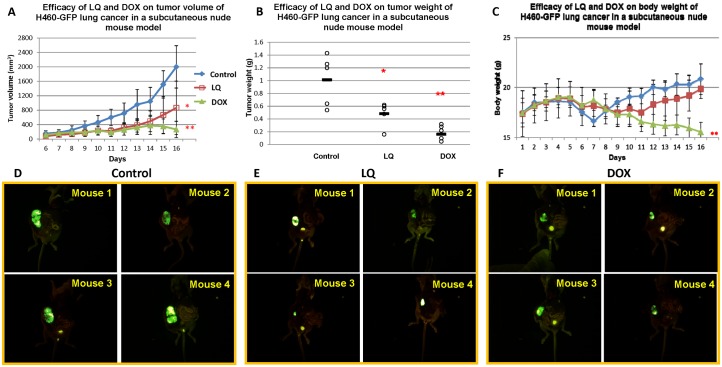
Efficacy of LQ on tumor volume (A), tumor weight (B) and body weight (C) in subcutaneous mouse tumor models of H460-GFP. Five mice were used in each group. After the subcutaneous tumors grew to 100 mm^2^, the nude mice were given either DOX (7.5 mg/kg i.v.) (twice a week for 3 times). PBS (po), LQ (600 mg/kg daily po). Tumor size and body weight were measured every day. Tumor weight was measured at the endpoint. Statistical significance between groups was determined with the Student’s *t*-test. After LQ treatment, lung tumor growth and tumor weight were significantly inhibited (*P<*0.01), compared to the untreated control group. DOX treatment also resulted in a significant tumor growth and weight inhibition compared to the untreated controls (*P<*0.01). The DOX-treated mice had significant body-weight loss compared to the LQ-treated and untreated controls (*P<*0.01). *p<0.05; **p<0.01. Images of H460-GFP tumors in live mice from the subcutaneous nude-mouse model are shown for the different treatment groups, and untreated control (**D**); LQ (**E**); and DOX (**F**).

From the data last section; we used 600 mg/kg of LQ (daily gavage) on the H460 human lung cancer cell line. We tested the H460-GFP cells growing subcutaneously compared to the H460 parental cell line in nude mice treated with LQ. We used DOX (7.5 mg/kg i.v., twice a week for three times) as the positive control to compare with LQ. In the control group, mice were given PBS for oral administration. Tumor imaging (Figure 3D, E & F) and caliper measurement (Fig. 3A) showed that tumor growth was inhibited after LQ and DOX treatment (the P value = 0.029, P value = 0.005, respectively), compared to the control mice. Final tumor weight was also significantly inhibited after LQ treatment and DOX treatment compared to the PBS control (*p* = 0.037, *p* = 0.01 respectively.) (Fig. 3B). However, DOX resulted in a significant loss in body weight compared to the LQ-treated animals (*p* = 0.002) and to the untreated controls (p = 0.001) (Fig. 3C). In contrast, no significant body weight loss was found in the LQ group compared to the control (Fig. 3C). The data also suggested that H460-GFP and the H460 parental cell line showed no difference on growth in mice and response to LQ.

### Comparison of LQ and DOX efficacy on the H460-GFP human lung cancer orthotopic model

**Figure 4:**
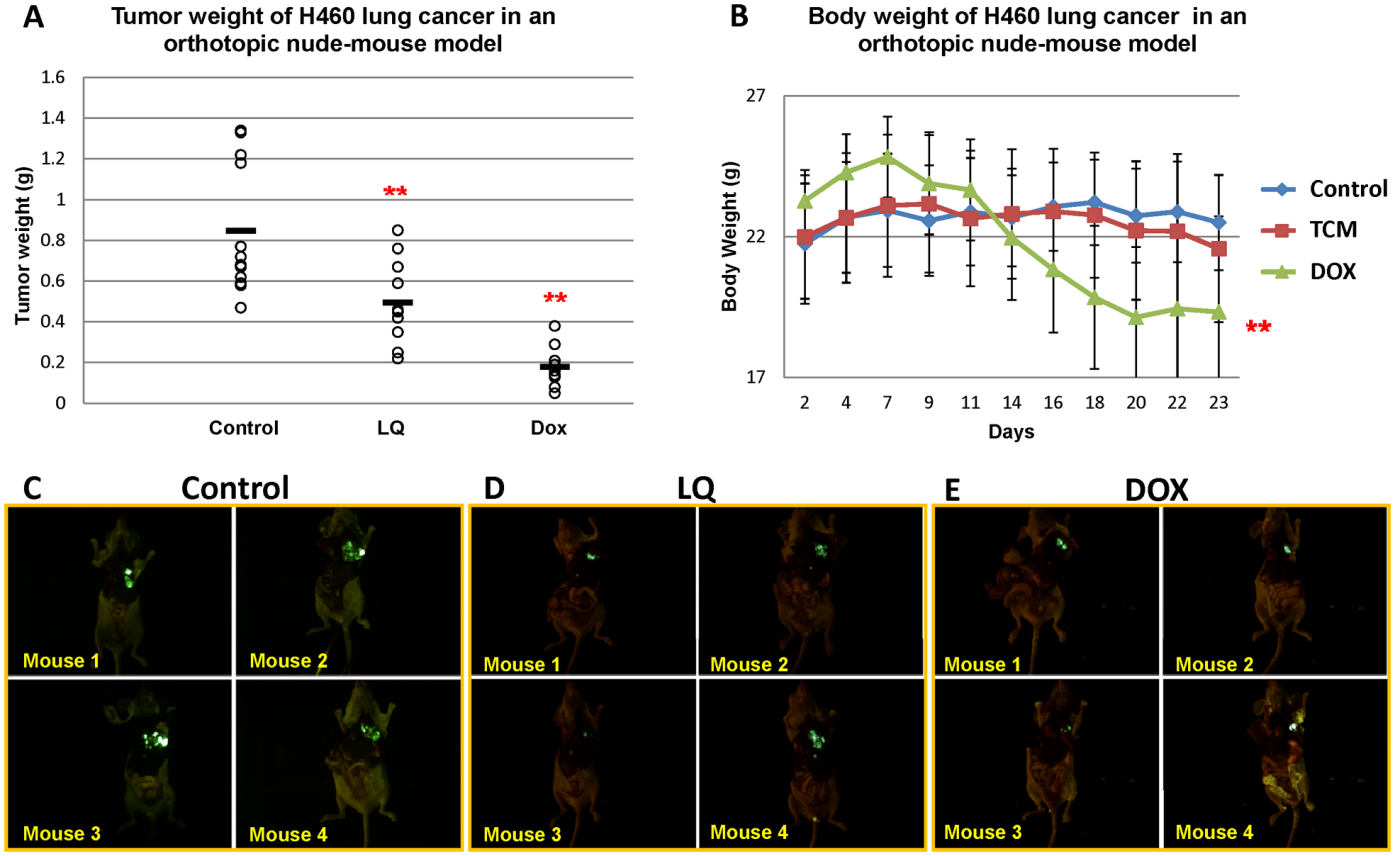
Efficacy of LQ on tumor volume, tumor weight (A) and body weight (B) in orthotopic H460-GFP lung cancer nude-mouse models. Ten mice were used in each group. H460-GFP fragments from tumors grown subcutaneously (1 mm in diameter) were harvested and implanted by surgical orthotopic implantation (SOI) into the left lungs of nude mice. The nude mice were given either PBS (po); DOX (7.5 mg/kg i.v. twice a week for 3 times from day 7) or LQ (600 mg/kg/day po daily. from day 7). Body weights were measured every day. Tumor weights were measured at the endpoint. Statistical significance between groups was determined with the Student’s *t-t*est. Lung tumor growth was significantly inhibited by LQ (*p<*0.01), without body weight loss. DOX inhibited lung tumor growth (*p<*0.001) but induced significant body weight loss compared to control mice (*p<*0.001). Open images of H460-GFP tumors in the orthotopic nude mouse model are shown for the different treatment groups and untreated control (**C**); LQ (**D**); and DOX (**E**).

To determine the anti-metastatic efficacy of LQ, an orthotopic nude-mouse model of human lung cancer, H460-GFP was used [12]. Open images of tumors in the lung from treated and untreated mice are shown in Fig. 4C–E. Tumor size was inhibited by LQ and DOX (all p<0.01) (Fig. 4A). Tumor weight was significantly decreased in the LQ-treated mice (600 mg/kg daily gavage) compared to the PBS control group (*p* = 0.0041) (Fig. 4A). DOX (7.5 mg/kg i.v., twice a week for three times) also inhibited tumor weight (*p<*0.0001) (Fig. 4A). DOX resulted in significant body weight loss compared to the untreated control (*p<*0.001) (Fig. 4B). In contrast, no significant body weight loss was found in the LQ-treated animals compared to the untreated control (Fig. 4B). There was no metastasis found in the control group.

The results above indicated LQ can significantly inhibit orthotopic lung tumor growth without toxicity unlike DOX which caused significant body weight loss.

### Efficacy of LQ on experimental lung cancer metastasis in nude mice and in immune-competent C57BL/6 mice

**Figure 5:**
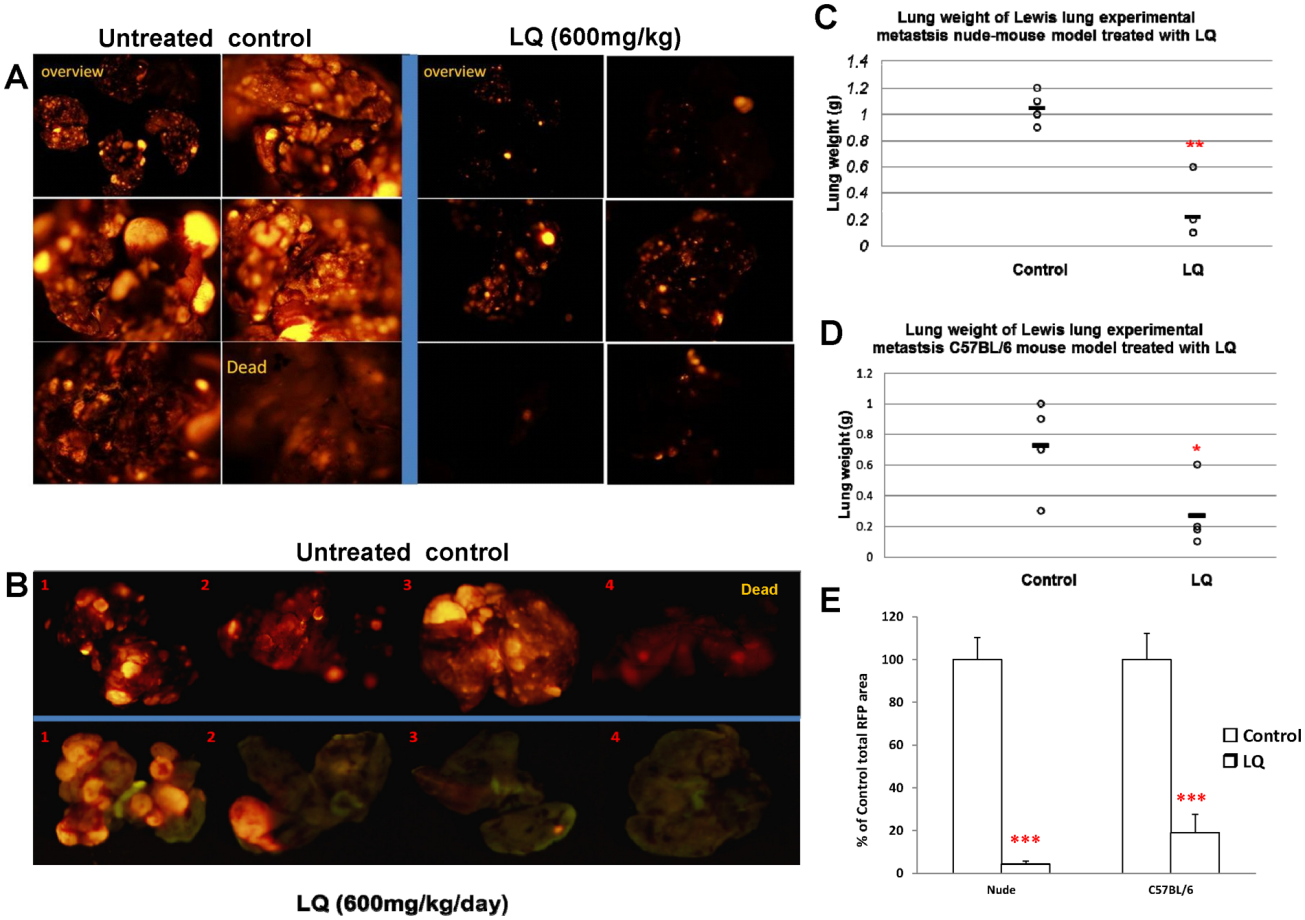
Efficacy of LQ on experimental lung metastasis. Six mice were used in each group. Individual images of excised lungs from the mice in the experimental metastasis model were obtained. LLC-RFP cancer cells (2×10^6^) were injected into the tail vein of nude mice or C57BL/6 mice. From day 2, the mice were given PBS (po) every day as the untreated control. Mice were treated with LQ (600 mg/kg/day po) for 10 days. The LQ-treated mice had significantly reduced lung weight (*p<*0.001) and reduced red fluorescence area in the lung (*p<*0.001) compared to the untreated PBS controls. **A** and **B** are images of the lungs. **C–D** are lung weights. **E** is the total red fluorescence area.

We tested the anti-metastatic efficacy of LQ in experimental mouse models. RFP-expressing Lewis lung carcinoma cells (LLC-RFP) cells were injected i.v. in both nude and C57BL/6 immune-competent mice in order to obtain experimental metastasis. Individual images of excised lungs from the mice were obtained. The lungs in the untreated control group were enlarged, and the color was dark red due to the size of the tumor. The LQ-treated animals had pinkish lungs with significantly reduced size compared to untreated controls. The red fluorescence intensity and area due to the presence of the LLC-RFP tumor was reduced in the LQ-treated mice compared to the untreated control mice (*p<*0.01) (Fig. 5A–B). LQ similarly inhibited tumor colonization in the lung in both nude and C57BL/6 mice (Fig. 5A–B). Reduced total lung weight in the LQ group, compared to the untreated control, also indicated reduced tumor colonization in both LQ-treated nude and C57BL/6 mice (*p* = 0.001 and *p* = 0.01, respectively) (Fig. 5C–D). The total area of red florescence of the lung was calculated. The fluorescent area correlated with lung weight. The total RFP tumor area in LQ-treated mice was 4% and 20%, compared to their nude or C57BL/6 control, respectively (all *p* value<0.001) (Fig. 5E). The results indicated that LQ can significantly inhibit the tumor colonization in the lung thereby indicating anti-metastatic efficacy.

### LQ prolonged survival of tumor-bearing animals

**Figure 6:**
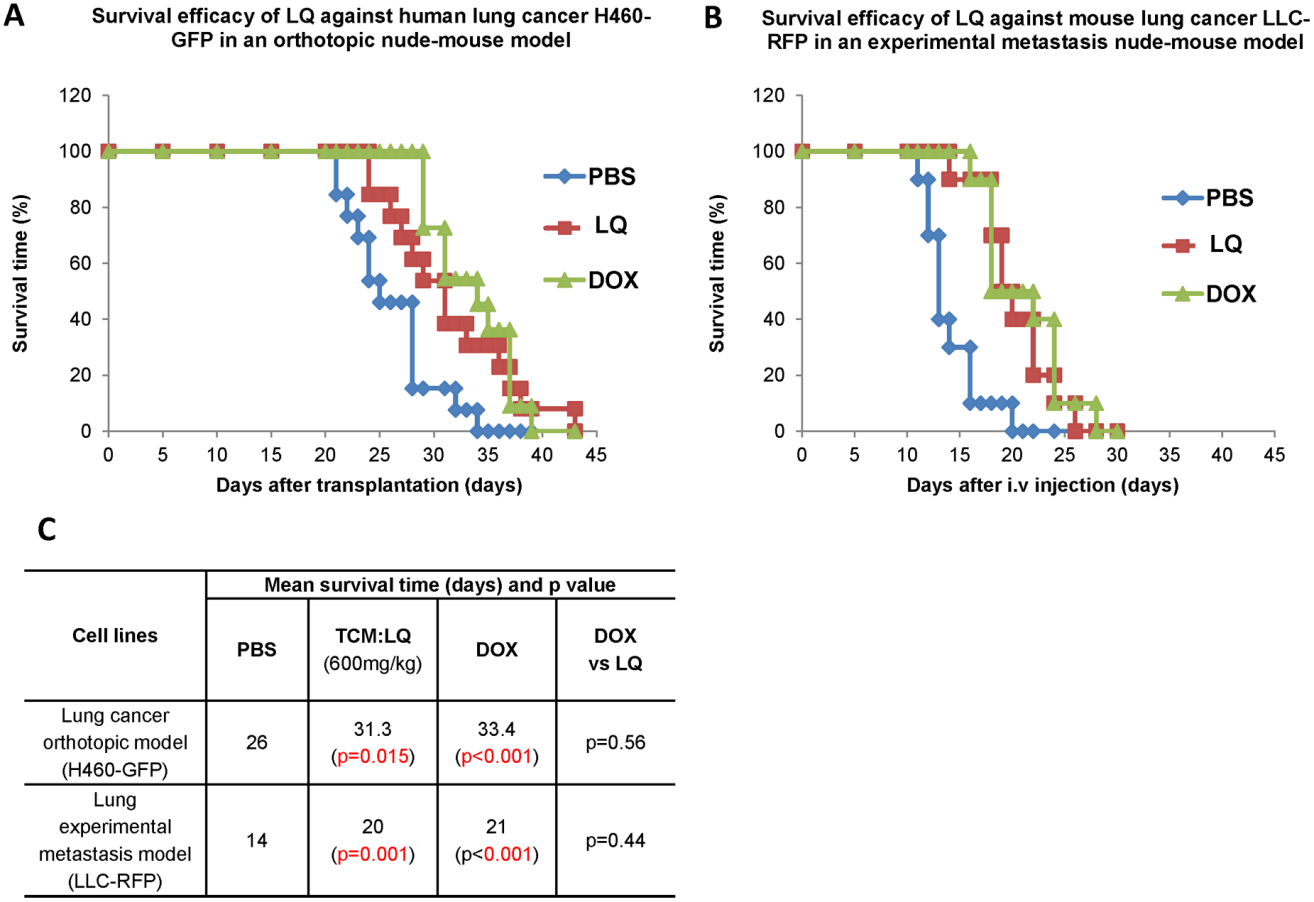
Survival curve of LQ-treated mice with H460-GFP orthotopic human lung cancer (A), and mice with LLC experimental lung metastasis (B). Ten mice were used in each group. LQ significantly increased survival in both the orthotopic (*p* = 0.015) and experimental metastasis (*p* = 0.001) models. DOX also significantly increased (*p<*0.001) survival in both models.

LQ significantly prolonged survival of mice with orthotopically-implanted H460 lung cancer as well as in mice with LLC experimental lung metastasis. Median-survival increased from 26 days in the untreated control animals to 30.3 days in mice treated with LQ (*p* = 0.005), to 33.4 days in mice treated with DOX in the orthotopic lung cancer model (*p* = 0.0001) (Fig. 6A, C). In the experimental metastasis model, median survival was increased from 14 days in the untreated control animals to 20 days in the LQ-treated mice (p = 0.001) and 22 days in the DOX-treated mice (*p<*0.001) (Fig. 6B, C). LQ prolonged survival of tumor-bearing animals comparable to DOX.

### Destruction of tumor blood vessels by LQ

**Figure 7:**
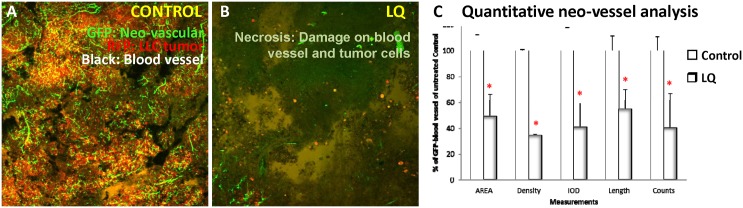
Effect of LQ on tumor blood vessels. LLC-RFP cells were grown in nestin-driven GFP (ND-GFP) transgenic nude mice in which nascent blood vessel expressed GFP. Three mice were used in each group. In the LQ-treated mice, tumor blood vessels appeared to be destroyed. The GFP florescence of blood vessels was quantitated for area, density, integrated optical density (IOD), length and counts. The measurements indicated that LQ decreased blood vessels by 50%.

In the ND-GFP mouse model, only new blood vessels induced by the tumor express nestin-driven GFP. Observation with the FV-1000 imaging system showed that blood vessels in the tumor were very dense and interwoven to form a blood vessel network (Fig. 7A). After LQ treatment, GFP blood vessels in LLC-RFP tumors in the ear and footpad were severely damaged (Fig. B). The GFP blood vessels fragmented in different areas of the tumor, and tumor necrosis was observed. Total fluorescent area, integrated optical intensity and length and density of GFP blood vessels were quantitated. The results showed that tumor blood vessels were reduced to ∼50% of control (*p<*0.05) after LQ administration (Fig. 7C).

### LQ induced cancer cell death *in vitro*

**Figure 8:**
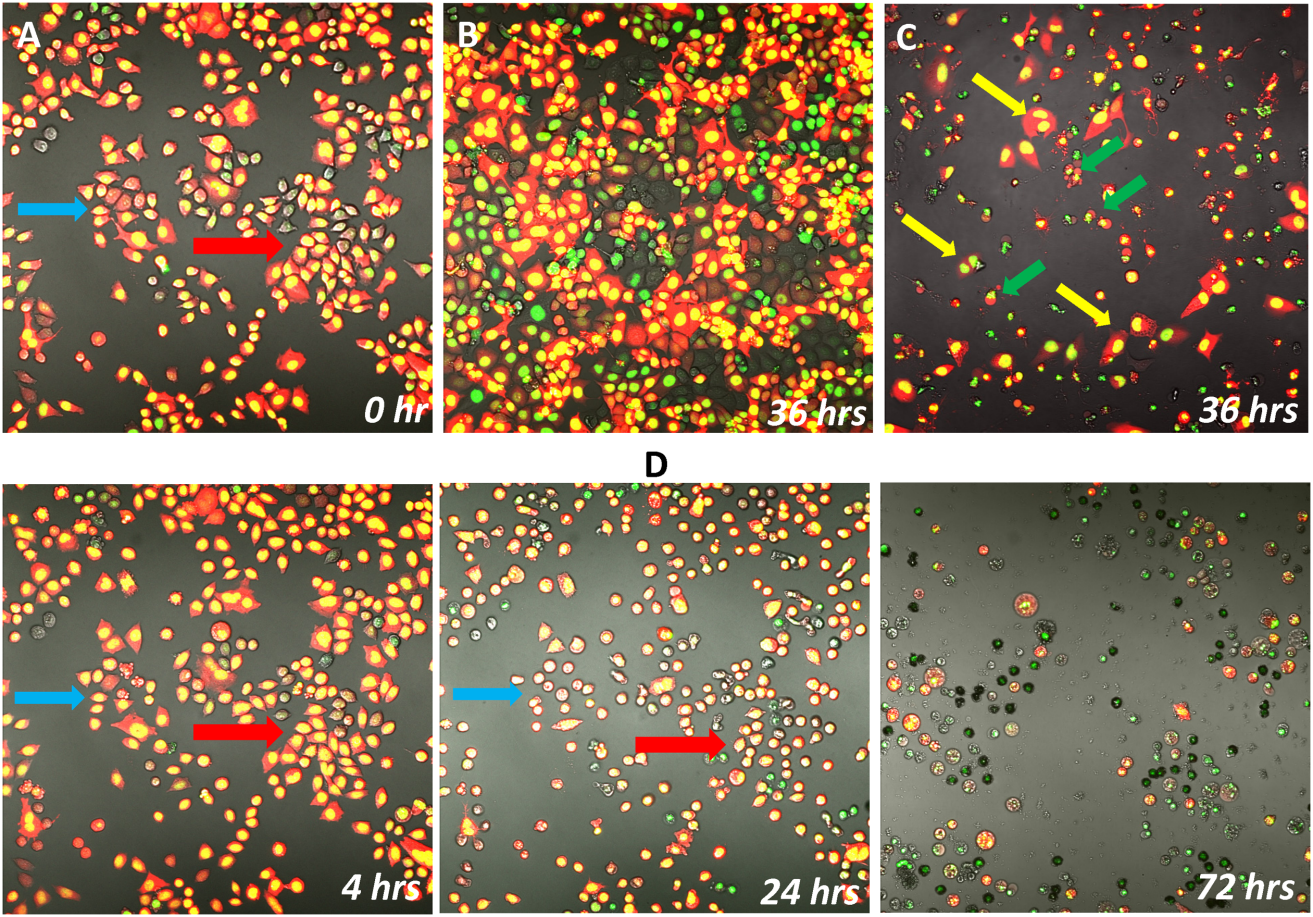
Time-Lapse imaging of H460 dual-color cells expressing GFP in the nucleus and RFP in the cytoplasm treated with LQ and CDDP. **A** and **B**) untreated control cells at 0 hour and 36 hours. **C**) CDDP-treated cells at 36 hours. **D**) time lapse imaging of LQ-treated H460 cells at 4, 24 and 72 hours. Cell proliferation was inhibited. Cell proliferation indicated by yellow arrows. Apoptotic cells indicated by green arrows. Blue and red arrows indicate changes in the same field.

H460 dual-color cells, expressing GFP in the nucleus and RFP in the cytoplasm were cultured in 35 mm peri-dish treated with LQ or CDDP. Cell proliferation was imaged every 4 hours for 72 hours. Cell proliferation was totally inhibited by LQ. After treatment, there was no mitosis. H460 dual-color cells treated with LQ decreased in size. The cytoplasm shrank at an early time point and disappeared, but the nuclei remained (Fig. 8A, B, D). At late-stages, nuclei fragmented. In the CDDP-treated H460 dual-color cells, apoptotic cells and few proliferating cells were observed (Fig. 8C). Approximately 60% of cells were apoptotic at the 36-hour time point. Fragmented nuclei were observed but had a different appearance than in cells treated with LQ. LQ may trigger a different cell death mechanism than apoptosis induced by CDDP. Further investigations will be conducted in the future.

### Comparison of efficacy of LQ and chemotherapy on cancer cell proliferation *in vitro*

**Figure 9:**
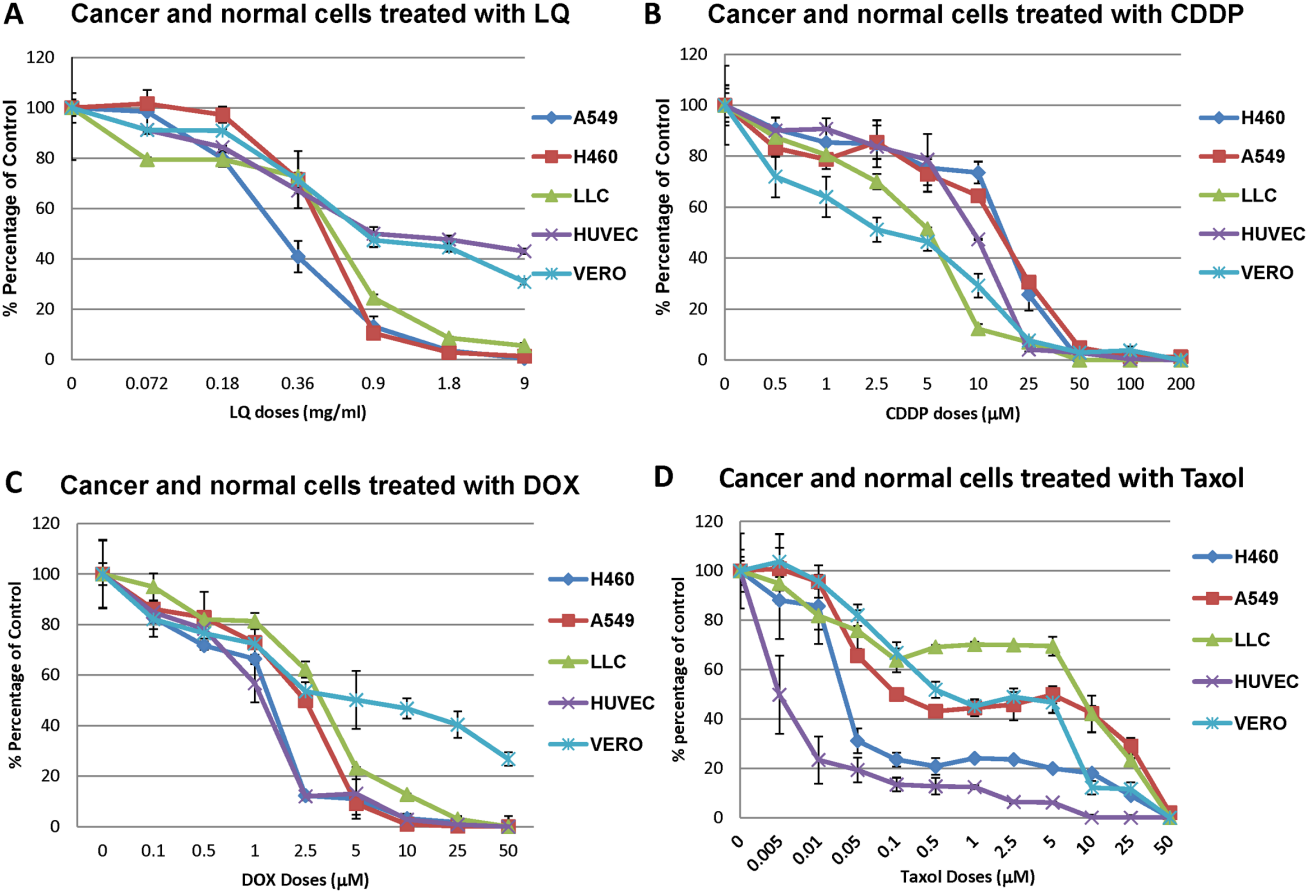
Efficacy of LQ on proliferation of cancer and normal cells *in vitro*. Cells were seeded in 96-well plates and incubated overnight. Cells were then exposed to LQ. H460, LLC and A549 were sensitive to LQ at 72 h (**A**). IC_50_ values were between 0.36∼0.45 mg/ml. All experiments showed a dose-dependent inhibition by LQ. However, LQ was less active on the normal cell lines HUVEC and VERO (**A**). The cancer and normal cells were all sensitive to DOX and CDDP (**B. C**). However, HUVEC and VERO cells were more sensitive to paclitaxol than the cancer cells (**D**). The graphs show combined values from two independent experiments, with each data point repeated in triplicate.

H460, A549, and LLC proliferation was tested *in vitro* with LQ and compared with CDDP, DOX, and paciltaxel (Fig. 9A–D). All cell lines were sensitive to LQ in a similar pattern. The IC_50_ were between 0.36 mg/ml to 0.45 mg/ml (Fig. 9A). H460, A549, and LLC proliferation was also inhibited by CDDP, paciltaxel, and DOX (Fig. 9B–D) with IC_50_ between 5∼25 µM, 1∼5 µM and 5∼25 µM, respectively. However, normal cell lines HUVEC and VERO were resistant to LQ with an IC_50_ approximately 9 mg/ml (more than 20-fold less sensitive than the cancer cells). In contrast, HUVEC and VERO were sensitive to CDDP comparable with cancer cells. HUVEC was sensitive to DOX comparable to cancer cells. However, VERO was 5-fold less sensitive to DOX than the cancer cells. HUVEC and VERO were more sensitive to paclitaxel than the cancer cells with the IC_50_ between 0.005∼0.01 µM (more than 100-fold more sensitive than the cancer cells). In order to rule out apparent efficacy due to pH change with LQ in the cell culture medium, the pH value at different concentrations of LQ was determined. The pH of RPMI 1640 medium with 10% FBS (no LQ), 9 mg/ml LQ or 1.8 mg/ml LQ was 7.61, 7.51, and 7.6, respectively. We also tested H460-dual color lung cancer cell proliferation in RPMI 1640 medium with 10% FBS at pH 7.51 using the MTT assay or live time imaging. The growth curve and growth pattern of cells at pH 7.51 was not significantly different from growth in RPMI 1640 medium with 10% FBS (pH 7.61) (Figure S1).

In the present report, we compared the efficacy of LQ against lung cancer in mouse models with DOX and CTX. LQ inhibited tumor size and weight comparable to cytotoxic chemotherapy but very importantly, without apparent toxicity. Survival of tumor-bearing mice was also prolonged by LQ, comparable to DOX. LQ had anti-metastatic efficacy observed by decreased cancer colonies in the lung. LQ also had anti-angiogenic activity as observed in lung tumors growing in ND-GFP transgenic nude mice, which selectively express GFP in nascent blood vessels. *In vitro*, death of lung cancer cells are induced by LQ comparable to cytotoxic chemotherapy. LQ was more potent to induce death in cancer cells than normal cells, unlike cytotoxic chemotherapy. The results of the present study indicate that LQ has non-toxic efficacy against metastatic lung cancer. Future studies should test LQ and other TCM in combination with cytotoxic chemotherapy in appropriate mouse models as a prelude to clinical studies. In addition, further experiments will test the antitumor and anti-metastatic efficacy of each herb separately in appropriate animal models.

## Supporting Information

Figure S1 (**A–C**) The growth curve of lung cancer cells growth in complete medium (CM) with different pH values. (**D**) The images of H460-Dual color cells growth in CM in pH 7.61 and pH 7.51.
(TIF)

## References

[1] Siegel R, Naishadham D, Jemal A (2012) Cancer statistics, 2012. CA: A Cancer J Clin 62: 10–29. 10.3322/caac.20138 22237781

[2] Newman DJ, Cragg GM (2012) Natural products as sources of new drugs over the 30 years from 1981 to 2010. J Natural Prod 75: 311–335. 10.1021/np200906s PMC3721181 22316239

[3] Zou YH, Liu XM (2003) Effect of astragalus injection combined with chemotherapy on quality of life in patients with advanced non-small cell lung cancer. Chinese J Int Trad Western Med 23: 733–735. 14626183

[4] Wang M, Zhang X, Xiong X, Yang Z, Sun Y, et al (2012) Efficacy of the Chinese traditional medicinal herb Celastrus orbiculatus Thunb on human hepatocellular carcinoma in an orthothopic fluorescent nude mouse model. Anticancer Res 32: 1213–1220. 22493351

[5] Hu M, Zhao M, An C, Yang M, Li Q, et al (2012) Real-time imaging of apoptosis induction of human breast cancer cells by the traditional Chinese medicinal herb tubeimu. Anticancer Res 32: 2509–2514. 22753707

[6] Zhang L, Wu C, Zhang Y, Liu F, Zhao M, et al (2013) Efficacy comparison of traditional Chinese medicine LQ versus gemcitabine in a mouse model of pancreatic cancer. J Cell Biochem 114: 2131–2137. 23553901 10.1002/jcb.24561

[7] Qi F, Li A, Inagaki Y, Gao J, Li J, et al (2010) Chinese herbal medicines as adjuvant treatment during chemo- or radio-therapy for cancer. Bioscience Trends 4: 297–307. 21248427

[8] Gai RY, Xu HL, Qu XJ, Wang FS, Lou HX, et al (2008) Dynamic of modernizing traditional Chinese medicine and the standards system for its development. Drug Discoveries & Therapeutics 2(1): 2–4. 22504447

[9] Wong R, Sagar CM, Sagar SM (2001) Integration of Chinese medicine into supportive cancer care: a modern role for an ancient tradition. Cancer Treatment Reviews 27: 235–246. 11545543 10.1053/ctrv.2001.0227

[10] Katz MH, Takimoto S, Spivack D, Moossa AR, Hoffman RM, et al (2003) A novel red fluorescent protein orthotopic pancreatic cancer model for the preclinical evaluation of chemotherapeutics. J Surg Res 113: 151–160. 12943825 10.1016/s0022-4804(03)00234-8

[11] Zhao M, Suetsugu A, Ma H, Zhang L, Liu F, et al (2012) Efficacy against lung metastasis with a tumor-targeting mutant of Salmonella typhimurium in immunocompetent mice. Cell Cycle 11: 187–193. 22186786 10.4161/cc.11.1.18667 PMC3272237

[12] Yang M, Hasegawa S, Jiang P, Wang X, Tan Y, et al (1998) Widespread skeletal metastatic potential of human lung cancer revealed by green fluorescent protein expression. Cancer Res 58: 4217–4221. 9766640

[13] Wang X, Fu X, Hoffman RM (1992) A patient-like metastasizing model of human lung adenocarcinoma constructed via thoracotomy in nude mice. Anticancer Res 12: 1399–1402. 1444197

[14] Rashidi B, Yang M, Jiang P, Baranov E, An Z, et al (2000) A highly metastatic Lewis lung carcinoma orthotopic green fluorescent protein model. Clin Exp Metastasis 18: 57–60. 11206839 10.1023/a:1026596131504

[15] Martínez-Gutierrez M, Castellanos JE, Gallego-Gómez JC (2011) Statins reduce dengue virus production via decreased virion assembly. Intervirology 54: 202–216. 21293097 10.1159/000321892

[16] Unger RE, Peters K, Sartoris A, Freese C, Kirkpatrick CJ (2014) Human endothelial cell-based assay for endotoxin as sensitive as the conventional Limulus Amebocyte Lysate assay. Biomaterials. pii: S0142-9612(13)01543-3. doi:10.1016/j.biomaterials.2013.12.059. [Epub ahead of print]. 10.1016/j.biomaterials.2013.12.059 24456607

[17] Hoffman RM, Yang M (2006) Subcellular imaging in the live mouse. Nature Protoc 1: 775–782. 17406307 10.1038/nprot.2006.109

[18] Hoffman RM, Yang M (2006) Color-coded fluorescence imaging of tumor-host interactions. Nature Protoc 1: 928–935. 17406326 10.1038/nprot.2006.119

[19] Hoffman RM, Yang M (2006) Whole-body imaging with fluorescent proteins. Nature Protoc 1: 1429–1438. 17406431 10.1038/nprot.2006.223

[20] Yamamoto N, Jiang P, Yang M, Xu M, Yamauchi K, et al (2004) Cellular dynamics visualized in live cells in vitro and in vivo by differential dual-color nuclear-cytoplasmic fluorescent-protein expression. Cancer Res 64: 4251–4256. 15205338 10.1158/0008-5472.CAN-04-0643

[21] Jiang P, Yamauchi K, Yang M, Tsuji K, Xu M, et al (2006) Tumor cells genetically labeled with GFP in the nucleus and RFP in the cytoplasm for imaging cellular dynamics. Cell Cycle 5: 1198–1201. 16760659 10.4161/cc.5.11.2795

[22] Hoffman RM (2005) The multiple uses of fluorescent proteins to visualize cancer in vivo. Nature Reviews Cancer 5: 796–806. 16195751 10.1038/nrc1717

[23] Li L, Mignone J, Yang M, Matic M, Penman S, et al (2003) Nestin expression in hair follicle sheath progenitor cells. Proc Natl Acad Sci USA 100: 9958–9961. 12904579 10.1073/pnas.1733025100 PMC187900

[24] Amoh Y, Li L, Yang M, Moossa AR, Katsuoka K, et al (2004) Nascent blood vessels in the skin arise from nestin-expressing hair-follicle cells. Proc Natl Acad Sci USA 101: 13291–13295. 15331785 10.1073/pnas.0405250101 PMC516562

[25] Amoh Y, Yang M, Li L, Reynoso J, Bouvet M, et al (2005) Nestin-linked green fluorescent protein transgenic nude mouse for imaging human tumor angiogenesis. Cancer Res 65: 5352–5357. 15958583 10.1158/0008-5472.CAN-05-0821

[26] Hayashi K, Yamauchi K, Yamamoto N, Tsuchiya H, Tomita K, et al (2009) A color-coded orthotopic nude-mouse treatment model of brain-metastatic paralyzing spinal cord cancer that induces angiogenesis and neurogenesis. Cell Prof 42: 75–82. 10.1111/j.1365-2184.2008.00574.x PMC6496699 19143765

[27] Hoffman RM (1999) Orthotopic metastatic mouse models for anticancer drug discovery and evaluation: a bridge to the clinic. Invest New Drugs 17: 343–359. 10759402 10.1023/a:1006326203858

[28] Astoul P, Colt HG, Wang X, Hoffman RM (1993) Metastatic human pleural ovarian cancer model constructed by orthotopic implantation of fresh histologically-intact patient carcinoma in nude mice. Anticancer Res 13: 1999–2002. 8297106

[29] Astoul P, Colt HG, Wang X, Hoffman RM (1994) A “patient-like” nude mouse model of parietal pleural human lung adenocarcinoma. Anticancer Res 14: 85–91. 8166461

[30] Astoul P, Colt HG, Wang X, Boutin C, Hoffman RM (1994) “Patient-like” nude mouse metastatic model of advanced human pleural cancer. J Cell Biochem 56: 9–15. 7806595 10.1002/jcb.240560104

[31] Kimura H, Hayashi K, Yamauchi K, Yamamoto N, Tsuchiya H, et al (2010) Real-time imaging of single cancer-cell dynamics of lung metastasis. J Cell Biochem 109: 58–64. 19911396 10.1002/jcb.22379

[32] Liu F, Zhang L, Hoffman RM, Zhao M (2010) Vessel destruction by tumor-targeting Salmonella typhimurium A1-R is enhanced by high tumor vascularity. Cell Cycle 9: 4518–4524. 21135579 10.4161/cc.9.22.13744 PMC3048048

[33] Yamauchi K, Yang M, Jiang P, Xu M, Yamamoto N, et al (2006) Development of real-time subcellular dynamic multicolor imaging of cancer-cell trafficking in live mice with a variable-magnification whole-mouse imaging system. Cancer Res 66: 4208–4214. 16618743 10.1158/0008-5472.CAN-05-3927

[34] Zhang QY, Zhao WH, Lai YJ (2005) Effect on late-stage mammary cancer treated by endocrinotherapy or chemotherapy combined with pingxiao capsule. Chinese J Integrated Trad Western Med 25: 1074–1076. 16398425

[35] Ottewell PD, Mönkkönen H, Jones M, Lefley DV, Coleman RE, et al (2008) Antitumor effects of doxorubicin followed by Zoledronic acid in a mouse model of breast cancer. J Natl Cancer Inst 100: 1167–1178. 18695136 10.1093/jnci/djn240

