# Supplementary figures and images for: Comparison of Efficacy and Toxicity of Traditional Chinese Medicine (TCM) Herbal Mixture LQ and Conventional Chemotherapy on Lung Cancer Metastasis and Survival in Mouse Models
Source: PLoS One. 2014 Oct 6;9(10):e109814. doi: 10.1371/journal.pone.0109814 (PMC4186882; doi:10.1371/journal.pone.0109814)

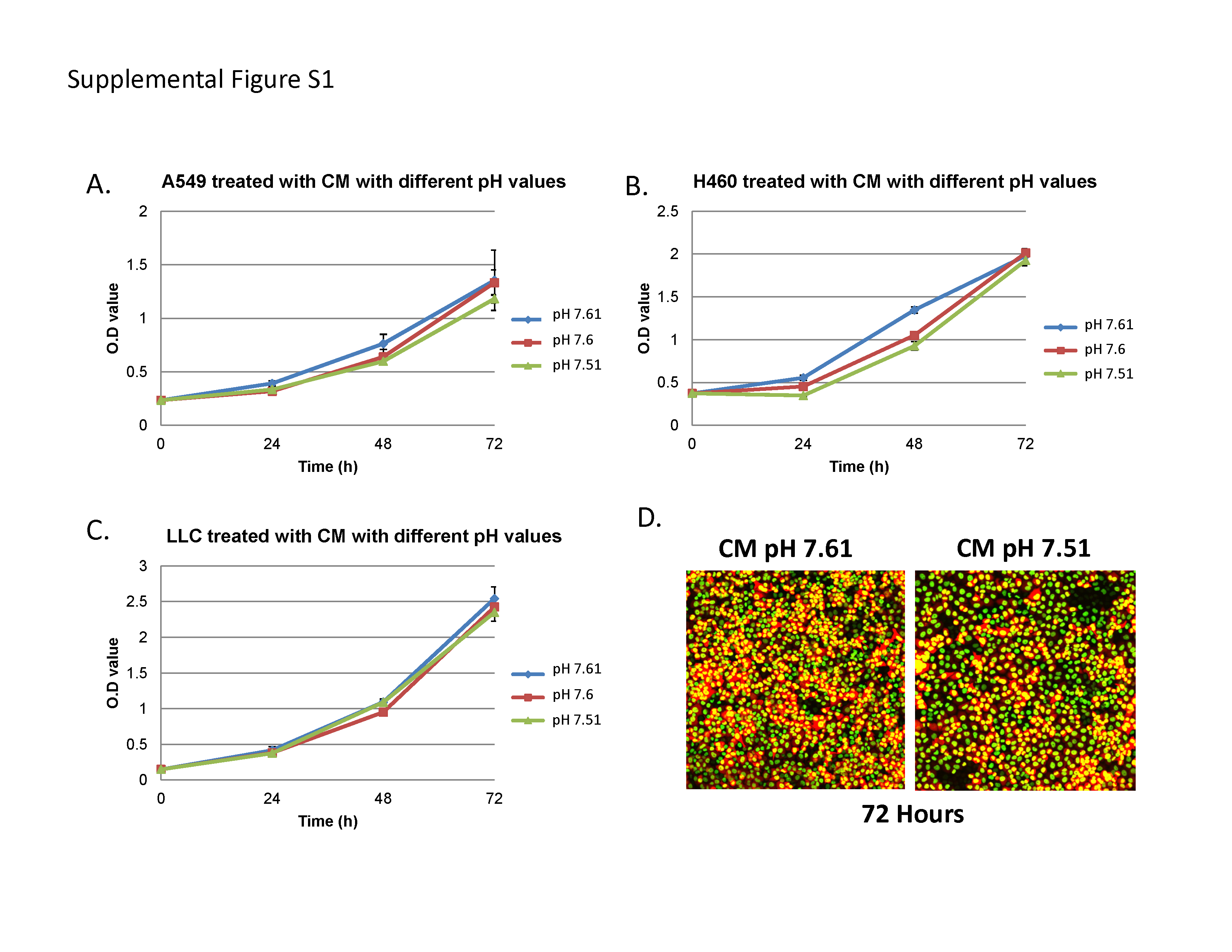

Supplement: Figure S1 — (A–C) The growth curve of lung cancer cells growth in complete medium (CM) with different pH values. (D) The images of H460-Dual color cells growth in CM in pH 7.61 and pH 7.51. (TIF) [file pone.0109814.s001.tif]
